# Label propagation method based on bi-objective optimization for ambiguous community detection in large networks

**DOI:** 10.1038/s41598-019-46511-2

**Published:** 2019-07-10

**Authors:** Junhai Luo, Lei Ye

**Affiliations:** School of Information and Communication Engineering, University of Electronic Science and Technology of China, Chengdu, China

**Keywords:** Computer science, Complex networks

## Abstract

Community detection is of great significance because it serves as a basis for network research and has been widely applied in real-world scenarios. It has been proven that label propagation is a successful strategy for community detection in large-scale networks and local clustering coefficient can measure the degree to which the local nodes tend to cluster together. In this paper, we try to optimize two objects about the local clustering coefficient to detect community structure. To avoid the trend that merges too many nodes into a large community, we add some constraints on the objectives. Through the experiments and comparison, we select a suitable strength for one constraint. Last, we merge two objectives with linear weighting into a hybrid objective and use the hybrid objective to guide the label update in our proposed label propagation algorithm. We perform amounts of experiments on both artificial and real-world networks. Experimental results demonstrate the superiority of our algorithm in both modularity and speed, especially when the community structure is ambiguous.

## Introduction

A variety of complex systems can be represented as networks, such as neural networks, social networks, and communication networks^1^. The nodes in networks represent the independent individuals in systems, while the edges represent the relations between them. In the community structure of networks, links within communities are dense while links between them are sparse. As an upstream task, community detection can be beneficial to other research, such as identifying top spreaders in social networks^2^, studying functional differences in brain networks^3^ and failure recovery in communication networks^4^.

Many efforts have been made for detecting community in networks, including hierarchical clustering algorithms^5–8^, spectral algorithms^9–11^, dynamic methods^12–17^, methods based on statistical inference^18–21^, modularity optimization algorithms^22–24^, and so on. It is worth pointing out that many existing detection methods suffer from their high time-complexity and cannot be applied to large networks. The label propagation algorithm (LPA) proposed by Raghavan *et al*. has proven to be near linear time-complexity for community detection^25^. LPA updates the label of every node with the most frequent label from its neighbors’. Although the update rule has small computational cost, it limits the accuracy of LPA.

In the past decade, many label propagation algorithms with different label update rules have been proposed to improve accuracy^26–28^. Similarly, they all have quite fast speed, because those label update rules are all based on local information, such as nodes’ degree, local density, and neighbors. Nonetheless, when the size of networks increases or the community structure becomes ambiguous, the accuracy of these methods still needs to be improved.

In this paper, we propose a new label propagation algorithm based on bi-objective optimization for detecting community. The algorithm initially assigns unique labels to all nodes and then iteratively updates the labels until the algorithm converges or specified iterations. Our algorithm not only converges faster but also performs better when the community structure is ambiguous, especially in large-scale networks.

The rest of the paper is organized as follows. In Section 2, we will review related works about community detection and label propagation. In Section 3, our proposed algorithm (LPAh) is described in details. In Section 4, we fully demonstrate the experimental results on artificial and real-world networks and analyze results in detail to illustrate the superiority of our approach.

## Related works

### Local clustering coefficient

In the unweighted undirected graph, an open triplet consists of three nodes that are connected by two edges and a closed triplet (i.e., triangle) consists of three nodes connected to each other^29^. The number of triangles on edge e_*ij*_ connects node *i* and node *j* is given as:

1 $${\tau }_{ij}=|{\rm{\Phi }}(i)\cap {\rm{\Phi }}(j)|,$$

where Φ(*i*) is the set of nodes immediately connected to node *i*. The number of triangles on node *i* is given as:

2 $${t}_{i}=\frac{1}{2}\sum _{j\in {\rm{\Phi }}(i)}{\tau }_{ij}.$$

The local clustering coefficient of one node is defined based on the triplet and measures the degree to which the node and its neighbors tend to cluster together^29^. The size of the set Φ(*i*) is given as *k*_*i*_, that is the degree of node *i*. The local clustering coefficient *C*_*i*_ of node *i* is defined as:

3 $${C}_{i}=\frac{{t}_{i}}{{k}_{i}\cdot ({k}_{i}-1)/2},$$

where t_i_ is the number of triangles on node *i* and k_i_(k_i_ − 1)/2 is the number of open triplets on node *i*.

### Evaluation for community partitions

A graph can be represented by its adjacency matrix *A* in which element *A*_*ij*_ is one when node *i* is connected to node *j*, and zero when not connected. The modularity compares the number of edges between nodes in the same community to the expected value in a null model^8^ and is formulated as:

4 $$Q=\frac{1}{2m}\sum _{i=1}^{n}\sum _{j=1}^{n}({A}_{ij}-\frac{{k}_{i}{k}_{j}}{2m})\delta (l(i),l(j))$$

where *m* is a total number of edges, *n* is the total number of nodes, *l*(*) is the community for the node * and *δ* is the Kronecker delta. The higher modularity indicates a better community partition, and the typical range of modularity is [0.3, 0.7]. Though modularity optimization methods suffer from resolution limit^30^, modularity is still a good metric for evaluating the quality of community partitions.

Normalized Mutual Information (NMI) is one of the widely used metrics that evaluate the quality of community partitions^31^. NMI can be used to compare the given partition with the ground-truth community partition. The closer to one the NMI is, the more similar the two partitions are.

### Label propagation

In general, label propagation algorithms initialize every node with unique labels and let the labels propagate through the network, that is, every node repeatedly updates its own label based on specific rules. Finally, nodes having the same labels compose one community.

In the LPA, one node selects the most frequent label from its neighbors’ as its new label^25^, and the rule can be expressed as:

5 $$l^{\prime} (v)=\mathop{\text{arg}\,\max }\limits_{l\in L}\sum _{u\in {\rm{\Phi }}(v)}\delta (l(u),l),$$

where *l*(u) is the current label of node *u*, *l*’(v) is the new label of node *v* and *L* is the set of labels for all nodes in the network. Barber and Clark reformulated the Eq. (5) in terms of the adjacency matrix *A* for the network^27^, giving:

6 $$l^{\prime} (v)=\mathop{\text{arg}\,\max }\limits_{l\in L}\sum _{u=1}^{n}{A}_{uv}\delta (l(u),l).$$

Barber and Clark also proposed a label propagation algorithm based on modularity (LPAm). LPAm considers the new label with constraining the sum of degrees of nodes in the same community, and its update rule is:

7 $$l^{\prime} (v)=\mathop{\text{arg}\,\max }\limits_{l\in L}(\sum _{u=1}^{n}{A}_{uv}\delta (l(u),l)-\lambda {k}_{v}{K}_{l}+\lambda {k}_{v}^{2}\delta (l(v),l)),$$

where

8 $${K}_{l}=\sum _{u=1}^{n}{k}_{u}\delta (l(u),l),$$

and the parameter λ is 1/2 m.

Later, Xie and Szymanski proposed a label propagation algorithm combining with the neighborhood (LPAc)^26^. The update rule of LPAc is:

9 $$l^{\prime} (v)=l(\mathop{\text{arg}\,\max }\limits_{{{\rm{\Phi }}}_{l}(v)}\{\sum _{u\in {{\rm{\Phi }}}_{l}(v)}(1+c\cdot {\tau }_{uv})\}),$$

where *Φ*_*l*_(*v*) is the set of nodes with the same label *l* and immediately connected to node *v*, c is the weight that controls the impact of neighbors and c belongs to [0, 1]. Usually, c = 1 performs better than other cases and Eq. (9) degrades into Eq. (5) when c = 0.

It is worth mentioning that the update process in label propagation can either be synchronous or asynchronous. In order to avoid the possible oscillations of labels, we focus our attention on the asynchronous update process here. Besides, when the current label of the updated node meets the update rule, algorithms always select a label at random from labels meet the update rule instead of keeping the current label.

### LFR benchmark networks

We test our algorithm and compare it with others on the artificial networks based on LFR benchmark^32^. In LFR benchmark, the mixing coefficient (*μ*) controls the expected fraction of edges between communities; the distribution of node degrees and community sizes follow the power law with exponent *γ* and *β*; the number of nodes is *n*; the average of node degrees is *kave*; the maximum of node degrees is *kmax*; the minimum of community sizes is *cmin* and the maximum of community sizes is *cmax*.

### Our approach

The local clustering coefficient measures the degree to which the local area tends to cluster together. The coefficient considers two factors: the number of edges connected to the node and the number of triangles on the node. Therefore, we try to optimize two objectives about both factors to detect the community structure.

The first objective is making the number of edges within communities as many as possible. The edge within communities means that two nodes connected by it belong to the same community.

The second objective is making the number of triangles within communities as many as possible. The triangle within communities means that three nodes that makeup it belongs to the same community.

We introduce a function H to roughly represent the linear combination of two objectives mentioned above as follows:

10 $$H=\sum _{v=1}^{n}\sum _{u=1}^{n}\{{A}_{uv}\delta (l(u),l(v))+{\alpha }_{1}\cdot {\tau }_{uv}{A}_{uv}\delta (l(u),l(v))\},$$

where the parameter *α*_1_ is a weight. Next, we can extract the term related to node *w* and rewrite function H as:

11 $$\begin{array}{c}H=\sum _{v\ne w}\sum _{u\ne w}(1+{\alpha }_{1}\cdot {\tau }_{uv}){A}_{uv}\delta (l(u),l(v))-(1+{\alpha }_{1}\cdot {\tau }_{ww}){A}_{ww}\\ \,+\,2\cdot \sum _{u=1}^{n}(1+{\alpha }_{1}\cdot {\tau }_{uw}){A}_{uw}\delta (l(u),l(w)).\end{array}$$

The third term of Eq. (11) can be regarded as a label update rule which can optimize two objectives. The rule can be denoted as:

12 $$l^{\prime} (v)=\mathop{\text{arg}\,\max }\limits_{l\in L}\sum _{u=1}^{n}\{{A}_{uv}\delta (l(u),l)+{\alpha }_{1}\cdot {\tau }_{uv}{A}_{uv}\delta (l(u),l)\},$$

In fact, Eq. (12) is a variant of Eq. (9). Obviously, when function H achieves the global maximum, all nodes have the same label, which is not a good community partition.

LPA assigns labels so as to make the number of edges within communities as many as possible. LPAm constrains the size of every community by Eq. (8), and at the same time, it increases the number of edges within communities.

Therefore, we firstly focus our attention on constraining the number of triangles within communities. The total number of triangles on nodes with the same label *l* is defined as:

13 $${T}_{l}=\frac{1}{2}\sum _{i=1}^{n}\sum _{j=1}^{n}{\tau }_{ij}{A}_{ij}\delta (l(i),l)=\sum _{i=1}^{n}{t}_{i}\delta (l(i),l).$$

The function for optimizing the number of triangles within communities is given as:

14 $$\begin{array}{rcl}{H}_{t} & = & \sum _{v=1}^{n}\sum _{u=1}^{n}{\tau }_{uv}{A}_{uv}\delta (l(u),l(v))-{\alpha }_{2}\cdot \sum _{l}{T}_{l}^{2}\\  & = & \sum _{v=1}^{n}\sum _{u=1}^{n}({\tau }_{uv}{A}_{uv}-{\alpha }_{2}\cdot {t}_{u}{t}_{v})\delta (l(u),l(v))\end{array}.$$

where α_2_ is the parameter that controls the strength of the constraint term. Similar to LPAm’s constraint about the number of edges within communities, α_2_ is selected as:

15 $${\alpha }_{2}=\varepsilon \frac{1}{{\rm{\Delta }}}$$

where Δ is the total number of triangles in a network and ε is a coefficient between 0 and 1. The suitable value for ε will be explained combined with experiments in Section 4. When the label of node v is updated, the label of v should be ignored to avoid its effect, that is

16 $${T^{\prime} }_{l}=\{\begin{array}{cc}{T}_{l}, & l\ne l(v)\\ {T}_{l}-{t}_{v}, & l=l(v)\end{array}.$$

From the relation between Eq. (10) and Eq. (12), the update rule corresponds to *H*_t_ is given as:

17 $$\begin{array}{rcl}l^{\prime} (v) & = & \mathop{\text{arg}\,\max }\limits_{l\in L}\sum _{u=1}^{n}({\tau }_{uv}{A}_{uv}-{\alpha }_{2}\cdot {t}_{u}{t}_{v})\delta (l(u),l)\\  & = & \mathop{\text{arg}\,\max }\limits_{l\in L}(\sum _{u=1}^{n}{\tau }_{uv}{A}_{uv}\delta (l(u),l)-{\alpha }_{2}{t}_{v}{{T}_{l}}^{^{\prime} })\end{array}.$$

The label propagation algorithm based on Eq. (17) is donated as LPAt.

Finally, the update rule of the label propagation algorithm that optimizes both objectives is formulated as:

18 $$l^{\prime} (v)=\mathop{\text{arg}\,\max }\limits_{l\in L}(\sum _{u=1}^{n}(1+{\alpha }_{1}{\tau }_{uv}){A}_{uv}\delta (l(u),l)-\lambda {k}_{v}{K^{\prime} }_{l}-{\alpha }_{1}{\alpha }_{2}{t}_{v}{T^{\prime} }_{l}),$$

where

19 $${K}_{l}^{\prime} =\{\begin{array}{ll}{K}_{l}, & l\ne l(v)\\ {K}_{l}-{k}_{v}, & l=l(v)\end{array}.$$

**Figure 1:**
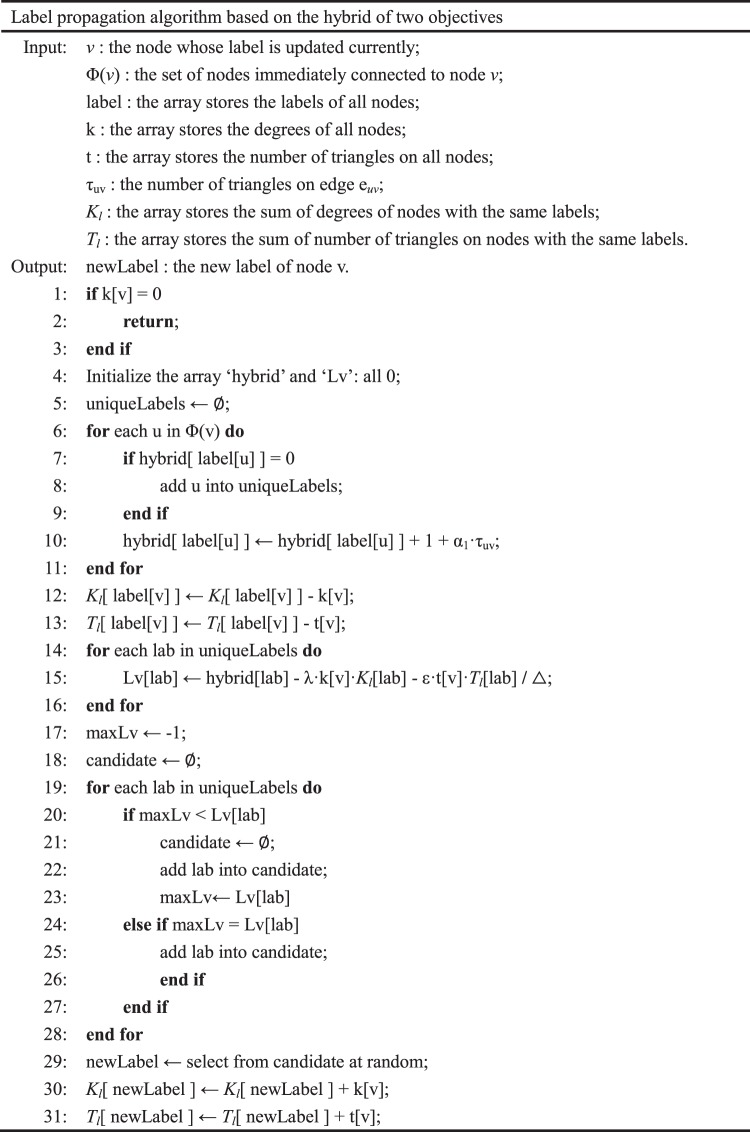
The main label propagation algorithm based on the hybrid of two objectives.

We donate the algorithm that optimizes both objectives as LPAh. In fact, we can conclude that LPAh performs better than LPAt through experiments. The main of LPAh is given in Fig. 1.

### Experiments and discussion

In this section, we test the LPAt and LPAh on artificial networks and real-world networks and compare their performance with LPA, LPAm, LPAc, CNM^5^, Louvain^33^ and G-CN. Among them, G-CN is one of the state-of-the-art methods^34^ for community detection; CNM and Louvain are popular community detection algorithms, and their time complexity are O(nlog^2^n) and O(m) respectively.

### The selection for ε

**Figure 2:**
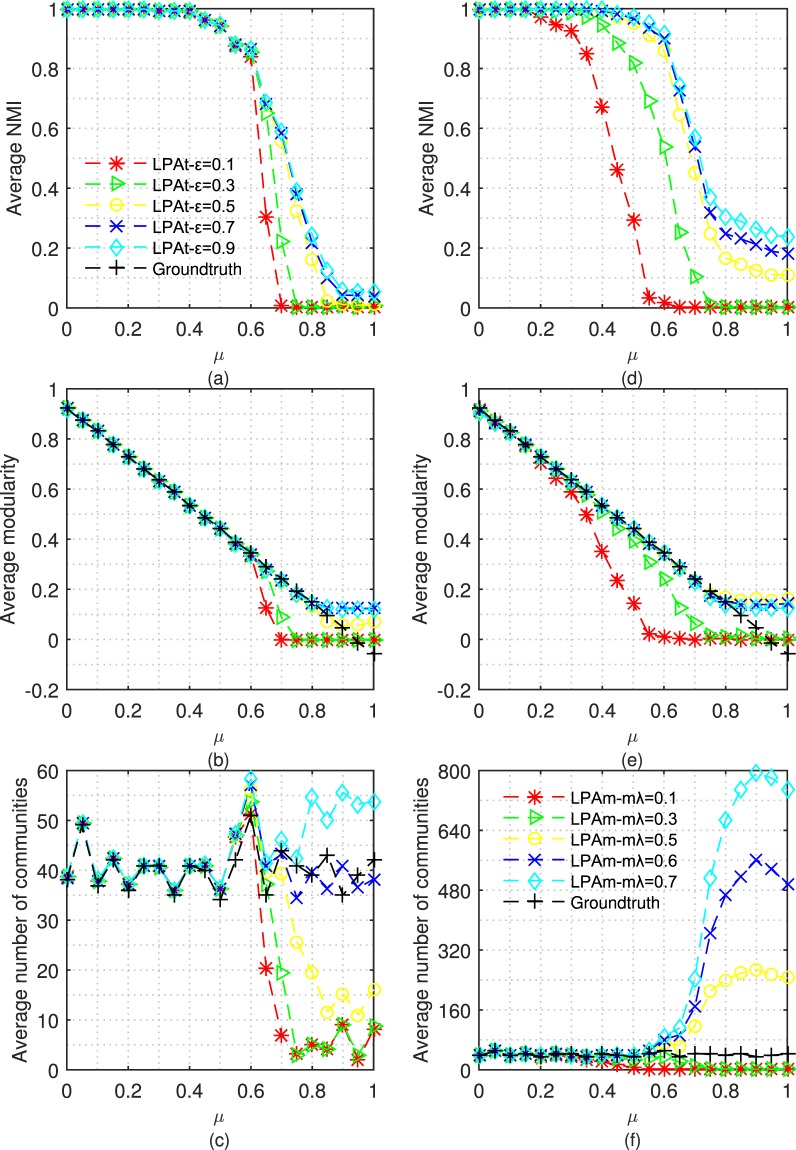
Tests of LPAt and LPAm with different strength of constraint on LFR benchmark networks: (**a–c**) and (**d–f**) show the results of LPAt and LPAm respectively. The parameters of LFR benchmark networks are: *μ* = 0 ~ 1, n = 5000, *kave* = 20, *kmax* = 0.1n, *γ* = −2, *β* = −1, *cmin* = 10, *cmax* = 0.1n.

The value of ε has a direct effect on the strength of the constraint term. Therefore, we test LPAt with different values of ε on LFR benchmark networks. For the purposes of comparison, we also test LPAm with different values of parameter mλ. Each algorithm doesn’t stop running until it converges or 20 iterations. Figure 2 shows the average of different metrics for performing LPAt and LPAm respectively 50 times on LFR benchmark networks.

Figure 2(a) shows the NMI of partitions given by LPAt. When the community structure is ambiguous (i.e., *μ* ≥ 0.6), with the increment of ε, the NMI values also increase, which means the partitions are closer to the ground-truth partitions. In Fig. 2(b), with the increment of ε, the increment of average modularity also demonstrates the quality of partitions becomes better. Figure 2(c) shows that when the community structure is ambiguous, the number of communities in partitions given by LPAt increases with the increment of ε.

The above observation also appears in Fig. 2(d~f). From the trend, we can conclude that when the community structure becomes ambiguous, if there is no or weak constraint, LPAt or LPAm tends to assign all nodes to a large community. However, when the constraint is strong, LPAt or LPAm tends to assign nodes into too many small communities. Therefore, a suitable value should be that the partitions given by LPAt or LPAm are as close as possible to the ground-truth partitions or the modularity is as large as possible.

As Barber and Clark gave, the suitable value of mλ is 0.5^27^. When mλ is larger than 0.5, the NMI and modularity have no obvious increment. It is worth pointing out that when mλ = 0.6 or 0.7, the NMI is slightly bigger than that when mλ = 0.5. This is because of the bias of NMI towards partitions with more communities^35^. Therefore, when mλ is larger than 0.5, the constraint tends to be excessive. Follow the above analysis, the suitable value for ε of LPAt approaches to 0.7.

Finally, we try to explain this idea mathematically. The triplet is a locally dense structure that contains more information than adjacent relationships. We can assign this information as weights to edges in the original network. The adjacency matrix of the new weighted network can be represented as:

20 $$W={[{w}_{ij}]}_{n\times n},$$

where

21 $${w}_{ij}={A}_{ij}\cdot {\tau }_{ij}.$$

The suitable value for mλ is inspired by the definition of modularity, that is, the constant term of Eq. (20):

22 $$\frac{\sum _{j}{A}_{ij}\cdot \sum _{i}{A}_{ij}}{\sum _{ij}{A}_{ij}}=\frac{1}{2}\cdot \frac{{k}_{i}\cdot {k}_{j}}{m}.$$

According to the definition of modularity in a weighted graph, the suitable value for ε should be 2/3 and determined by

23 $$\frac{\sum _{j}{w}_{ij}\cdot \sum _{i}{w}_{ij}}{\sum _{ij}{w}_{ij}}=\frac{2{t}_{i}\cdot 2{t}_{j}}{2\sum _{i}{t}_{i}}=\frac{2}{3}\cdot \frac{{t}_{i}\cdot {t}_{j}}{{\rm{\Delta }}}$$

Besides, from Fig. 2, we can conclude that LPAt with ε = 2/3 performs not better than LPAm with mλ = 0.5. Therefore, we focus our attention on LPAh with ε = 2/3.

### The selection for α_1_

**Figure 3:**
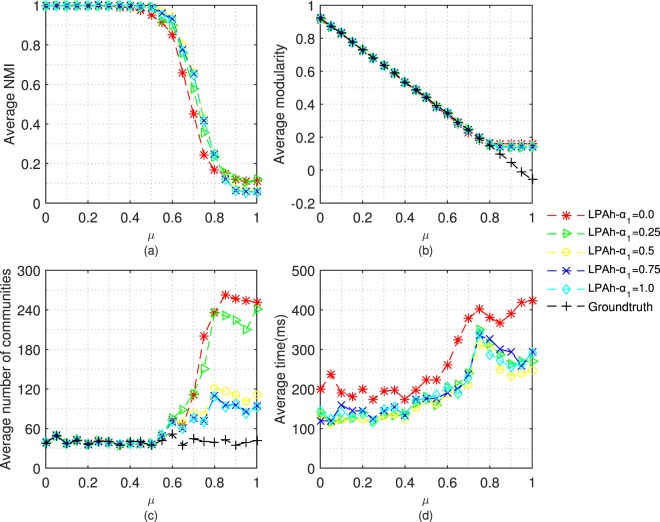
Tests of LPAh with different *α*_1_ on LFR benchmark networks. The parameters of LFR benchmark networks are: *μ* = 0 ~ 1, n = 5000, *kave* = 20, *kmax* = 0.1n, *γ* = −2, *β* = −1, *cmin* = 10, *cmax* = 0.1n.

Here, under ε = 2/3, we test LPAh with different values of *α*_1_ on LFR benchmark networks. The iteration time of the algorithm is also less than or equal to 20. The results of the above experiments are shown in Fig. 3.

As we can see from Fig. 3(a), the increment of *α*_1_ can improve the NMI of detection results. However, when *α*_1_ is between 0.5 and 1, the difference in the improvement is not obvious. Figure 3(b) shows that different *α*_1_ has no obvious effects on the modularity of detection results. In Fig. 3(c), when community structure is ambiguous, with the increment of *α*_1_, the number of communities that are detected by LPAh decreases. In fact, when *α*_1_ is 0, LPAh degrades into LPAm. From the discussion in section 4.1, the partition that assigns nodes into too many small communities means the constraint is strong. The execution time of LPAh under different values of *α*_1_ demonstrates the faster convergence when *α*_1_ is larger than 0. Considering LPAc often performs better when the weight c is 1, we also determine to select the *α*_1_ as 1.

### Comparison of artificial networks

In order to fully compare all algorithms, we not only consider the networks with different strength of community structure but also take the size of networks into account.

**Figure 4:**
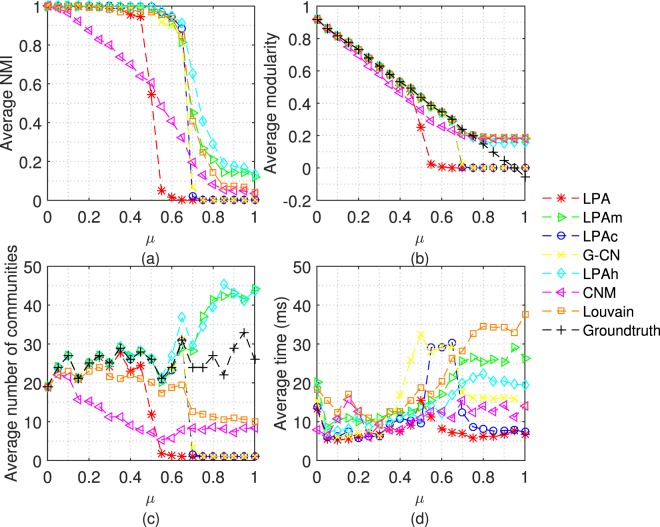
Tests of 7 algorithms on LFR networks with n = 1000. The parameters of LFR networks are: *μ* = 0 ~ 1, n = 1000, *kave* = 20, *kmax* = 0.1n, *γ* = −2, *β* = −1, *cmin* = 10, *cmax* = 0.1n.

**Figure 5:**
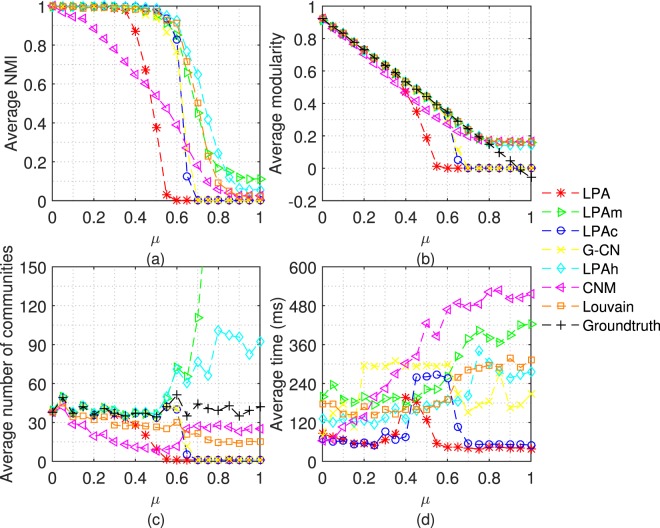
Tests of 7 algorithms on LFR networks with n = 5000. The parameters of LFR networks are: *μ* = 0 ~ 1, n = 5000, *kave* = 20, *kmax* = 0.1n, *γ* = −2, *β* = −1, *cmin* = 10, *cmax* = 0.1n.

**Figure 6:**
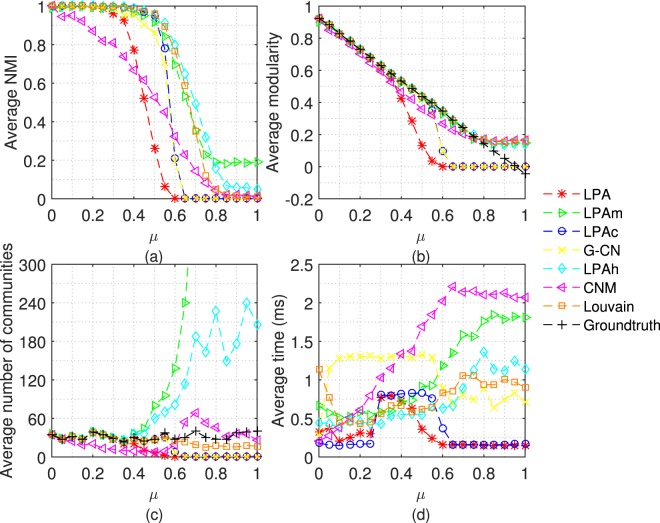
Tests of 7 algorithms on LFR networks with n = 10000. The parameters of LFR networks are: *μ* = 0 ~ 1, n = 10000, *kave* = 20, *kmax* = 0.1n, *γ* = −2, *β* = −1, *cmin* = 10, *cmax* = 0.1n.

Firstly, we test 7 algorithms on LFR networks with different mixing coefficient (*μ*). Each algorithm doesn’t stop running until it converges or 20 iterations. The average results achieved by performing each algorithm 50 times are shown in Figs 4, 5 and 6.

Before analyzing the results of experiments, we divide the variation range of *μ* into 3 parts to observe every figure: when 0 ≤ *μ* < 0.5, the most edges connect nodes belong to the same community, which means the community structure is clear; when 0.5 ≤ *μ* ≤ 0.65, the community structure is ambiguous because the modularity is still larger than 0.3; when *μ* > 0.65, the community structure is very weak.

Figure 4 shows the NMI, modularity, number of communities and execution time of 7 algorithms on LFR networks with 1000 nodes. As we can see from Fig. 4(c), when the community structure becomes ambiguous, LPA, LPAc and G-CN tend to assign all nodes into a large community, and the tendency of LPA appears earlier. Unlike them, LPAm and LPAh tend to assign nodes into many communities. Therefore, in Fig. 4(a,b), LPAh and LPAm both perform better than LPA, LPAc and G-CN. When the community structure is ambiguous (0.5 ≤ *μ* ≤ 0.65), LPAh performs better than LPAm both in NMI and modularity. Notice that, when the community structure is very weak (*μ* > 0.65), the modularity of LPAm and Louvain is slightly larger than that of LPAh which may be because LPAm and Louvain both aim at optimizing modularity. However, at this time, the modularity is lower than the typical value (0.3), and the slight superiority has no practical significance. Figure 4(d) shows the execution time of algorithms on different networks. Besides, for non-label propagation algorithm, CNM always performs not well and Louvain aggregate excessively (the average number of communities is lower than the ground-truth even if the community structure is clear).

From the experiments on the network with 5000 and 10000 nodes in Figs 5 and 6, we can get the conclusions consistent with the above.

In Figs 5(c) and 6(c), in order to exhibit the results of other algorithms clearly, we only plot part of the results of LPAm, because the number of communities detected by LPAm increases dramatically. We can compare the experimental results from a different perspective - under the same *μ* and different sizes of networks. Let’s focus our attention on the cases that the community structure is ambiguous, especially *μ* = 0.6 and 0.65. It is obvious that the accuracy of LPA, LPAc and G-CN decreases significantly, and even unable to detect the community structure. In the above cases, the accuracy of LPAh, LPAm, and Louvain only decrease slightly, and LPAh still performs better than LPAm. In terms of execution time, LPAh still performs quite well.

**Figure 7:**
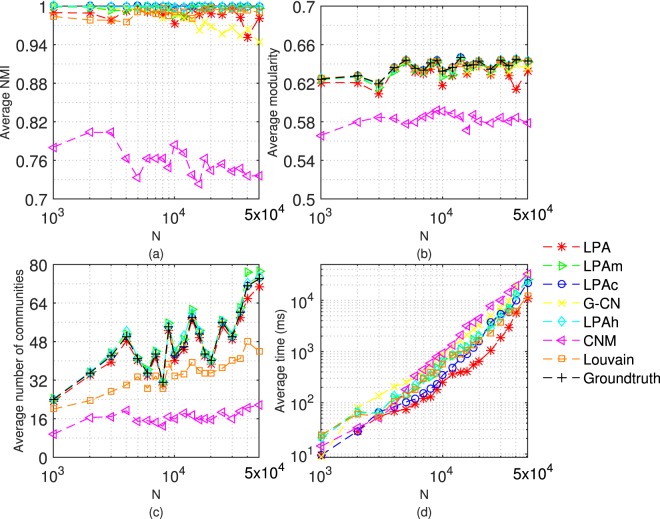
Tests of 7 algorithms on LFR networks with *μ* = 0.3. The parameters of LFR networks are: *μ* = 0.3, n = 1000~50000, *kave* = 20, *kmax* = 0.1n, *γ* = −2, *β* = −1, *cmin* = 10, *cmax* = 0.1n.

**Figure 8:**
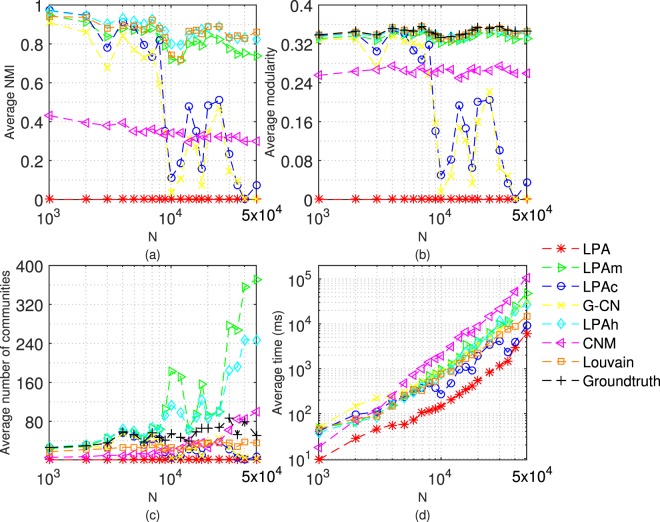
Tests of 7 algorithms on LFR networks with *μ* = 0.6. The parameters of LFR networks are: *μ* = 0.6, n = 1000~50000, *kave* = 20, *kmax* = 0.1n, *γ* = −2, *β* = −1, *cmin* = 10, *cmax* = 0.1n.

Next, we test 7 algorithms on LFR networks with different size, that is, the number of nodes (n) is 1000, 2000, 3000, 4000, 5000, 6000, 7000, 8000, 9000, 10000, 12000, 14000, 16000, 18000, 20000, 25000, 30000, 35000, 40000 and 50000. Here, we consider the situation in which the community structure is clear or ambiguous (*μ* = 0.3 or 0.6). Each algorithm doesn’t stop running until it converges or 20 iterations. The average results achieved by performing each algorithm 20 times are shown in Figs 7 and 8.

Figure 7 shows the performance of 7 algorithms on different sizes of networks when the community structure is clear (*μ* = 0.3). The algorithms based on label propagation perform better than CNM in NMI and modularity, and better than Louvain in the number of communities. According to the execution time, the time complexity of 7 algorithms is comparable and close to linear.

Compared to Fig. 7, the results in Fig. 8 are more interesting. Although LPA is fastest, it can’t find the community structure. With the increment of network size, the accuracy of LPAc and G-CN decreases significantly. In fact, as shown in Table 1, the detection results (red data) of LPAc and G-CN sometimes are still comparable to LPAh. In Table 1, when LPAc can’t detect the community structure, it will converge fast, which causes the fluctuations in the execution time of LPAc in Fig. 8(d). When n is larger than 10000, the performance of LPAm in NMI and modularity also decreases slightly. With the increment of network size, two algorithms with constraints, namely LPAm and LPAh, perform differently from other algorithms in the number of communities in Fig. 8(c).

**Table 1:** Tests of LPAc on LFR networks with *μ = *0.6. The parameters of LFR networks are: *μ = *0.6, n = 20000, *kave = *20, *kmax* = 0.1n, *γ* = -2, *β* = -1, *cmin* = 10, *cmax* = 0.1n.

t(ms)	iterations	c	NMI	Q
1468	8	5	0.0123	0.0016
638	5	4	0.0087	0.0010
2553	20	53	0.7949	0.3340
2527	20	54	0.8113	0.3395
2530	20	61	0.8518	0.3461
2510	20	46	0.7375	0.3123
2511	20	58	0.8400	0.3453
2506	20	57	0.8218	0.3403
2511	20	55	0.8007	0.3366
1384	11	3	0.0044	0.0005
626	5	3	0.0044	0.0005
1396	11	4	0.0087	0.0010
1759	14	5	0.0123	0.0016
2501	20	54	0.7980	0.3369
2520	20	56	0.8049	0.3380
2496	20	50	0.7617	0.3269
766	6	5	0.0123	0.0016
2496	20	50	0.7639	0.3283
879	7	4	0.0083	0.0010
2516	20	51	0.7657	0.3234

### Comparison of real-world networks

Finally, we run each algorithm on 7 real-world networks until it converges or 20 iterations. Because some networks do not have the ground-truth partitions, or some partitions are concluded by researchers, we only consider the average of modularity (Q), execution time (t) and number of communities (c). The detection results of all algorithms are shown in Table 2.

**Table 2:** Detection results on real-world networks.

network		Karate	Dolphins	Football	Facebook	ca-GrQc	ca-HepPh	cit-HepTh
n		34	62	115	4039	5242	12008	27770
m		78	159	613	88234	14484	118489	352285
c	LPA	2	3	11	56	724	656	580
	LPAc	2	4	14	24	720	818	843
	G-CN	3	4	14	25	682	814	834
	LPAm	7	9	13	98	1243	1397	1488
	LPAh	6	8	13	55	1066	1206	1444
	CNM	3	4	7	14	419	424	289
	Louvain	4	5	10	16	392	317	171
Q	LPA	0.307	0.474	0.586	0.813	0.793	0.455	0.488
	LPAc	0.363	0.527	0.565	0.732	0.797	0.534	0.590
	G-CN	0.315	0.527	0.562	0.738	0.800	0.550	0.584
	LPAm	0.345	0.500	0.581	0.813	0.709	0.589	0.569
	LPAh	0.363	0.515	0.585	0.821	0.752	0.602	0.589
	CNM	0.381	0.494	0.571	0.778	0.814	0.589	0.519
	Louvain	0.419	0.520	0.604	0.835	0.860	0.658	0.650
t (ms)	LPA	<1	<1	<1	206	187	867	4820
	LPAc	<1	<1	<1	240	196	987	5222
	G-CN	<1	<1	<1	263	220	1247	6632
	LPAm	<1	<1	<1	242	391	2199	8930
	LPAh	<1	<1	<1	239	218	2287	9736
	CNM	<1	<1	1.59	66	56	943	8581
	Louvain	<1	<1	1.68	197	144	853	7752

In Table 2, Karate^36^, Dolphins^37^, Football^38^ and Facebook^39^ network are social networks between persons or animals in different scenarios; ca-GrQc^40^ and ca-HepPh^40^ are collaboration networks; cit-HepTh^41^ is a citation network. According to the optimal results highlighted with red color in Table 2, though LPAh is not the clear winner, it performs well enough. The number of communities detected by LPAm and LPAh is larger than others, which is because of the constraint term in their objective function. The modularity of LPAh is comparable to that of other algorithms and even performs better on some networks. Because of Louvain and CNM aim at optimizing the modularity, Q detected by Louvain and CNM is sometimes larger than that by LPAh.

## Conclusion

We propose a new label propagation algorithm, LPAh, which is based on two optimization objectives. The algorithm performs well on large-scale networks, even if the community structure is ambiguous.

The optimization objective is inspired by the local clustering coefficient and has the constraint to avoid the trend that merges too many nodes into a large community. To select the suitable coefficient (ε) for the constraint, we test the algorithm with different strength of constraint on various artificial networks and compare the results. Under the selected parameter (ε), our algorithm performs better on LFR networks than other existing algorithms including the state of the art one, especially when the community structure is ambiguous. Besides, the experiments on various real-world networks also show the superiority of our algorithm in both modularity and speed.

## Supplementary information

Supplementary
