# Supplementary material for: Label propagation method based on bi-objective optimization for ambiguous community detection in large networks
Source: Sci Rep. 2019 Jul 10;9:9999. doi: 10.1038/s41598-019-46511-2 (PMC6620331; doi:10.1038/s41598-019-46511-2)
Supplement: Supplementary file 1 — Supplementary [file 41598_2019_46511_MOESM1_ESM.docx]

**Label propagation method based on bi-objective optimization for ambiguous community detection in large networks**

Junhai Luo*, Lei Ye

**Supplementary**

The objective function of Eq. (10) is rewritten as follows:

$H\text{=}\sum_{v=1}^{n} \sum_{u=1}^{n} \{A_{uv}\delta(l(u),l(v))+\alpha_{1}\cdot\tau_{uv}A_{uv}\delta(l(u),l(v))\}$. (S-1)

Here, A_uv_ is equal to 1 when node u is connected to node v, and zero when not connected. Therefore, only the edges within communities have contribution to H. Obviously, when all nodes have the same label, function H achieves the global maximum.

To avoid the meaningless global maximum, LPAh aims at optimizing the objective function H_h_:

$\begin{matrix} H_{h}=\sum_{v=1}^{n} \sum_{u=1}^{n} \{A_{uv}\delta(l(u),l(v))+\alpha_{1}\tau_{uv}A_{uv}\delta(l(u),l(v))\}-\lambda\sum_{l} K_{l}^{2}-\alpha_{1}\alpha_{2}\sum_{l} T_{l}^{2} \\ \text{=}\sum_{v=1}^{n} \sum_{u=1}^{n} \{A_{uv}\delta(l(u),l(v))\}-\lambda\sum_{l} K_{l}^{2}+\alpha_{1}[\sum_{v=1}^{n} \sum_{u=1}^{n} \{\tau_{uv}A_{uv}\delta(l(u),l(v))\}-\alpha_{2}\sum_{l} T_{l}^{2}] \end{matrix}$, (S-2)

where

$K_{l}=\sum_{i=1}^{n} \sum_{j=1}^{n} A_{ij}\delta(l(i),l)=\sum_{i=1}^{n} k_{i}\delta(l(i),l)$, (S-3)

$T_{l}\text{=}\frac{1}{2}\sum_{i=1}^{n} \sum_{j=1}^{n} \tau_{ij}A_{ij}\delta(l(i),l)=\sum_{i=1}^{n} t_{i}\delta(l(i),l)$. (S-4)

The Eq. (S-2) can be rewritten as:

$\begin{matrix} H_{h}=\sum_{v=1}^{n} \sum_{u=1}^{n} \{A_{uv}\delta(l(u),l(v))+\alpha_{1}\tau_{uv}A_{uv}\delta(l(u),l(v))\}-\lambda\sum_{l} K_{l}^{2}-\alpha_{1}\alpha_{2}\sum_{l} T_{l}^{2} \\ \text{=}\sum_{v=1}^{n} \sum_{u=1}^{n} (A_{uv}-\lambda k_{u}k_{v})\cdot\delta(l(u),l(v))+\alpha_{1}[\sum_{v=1}^{n} \sum_{u=1}^{n} (\tau_{uv}A_{uv}-\alpha_{2}t_{u}t_{v})\cdot\delta(l(u),l(v))] \end{matrix}$. (S-5)

Next, we can extract the term related to node *w* and rewrite function H_h_ as:

$\begin{matrix} H_{h}\text{=}\sum_{v=1}^{n} \sum_{u=1}^{n} \left\{(A_{uv}-\lambda k_{u}k_{v})\cdot\delta(l(u),l(v))\right\}+\alpha_{1}\sum_{v=1}^{n} \sum_{u=1}^{n} \left\{(\tau_{uv}A_{uv}-\alpha_{2}t_{u}t_{v})\cdot\delta(l(u),l(v))\right\} \\ \text{=}\sum_{v\neq w} \sum_{u\neq w} \left\{(A_{uv}-\lambda k_{u}k_{v})\cdot\delta(l(u),l(v))\right\}+2\sum_{u=1}^{n} \left\{(A_{uw}-\lambda k_{u}k_{w})\cdot\delta(l(u),l(w))\right\}-(0-\lambda k_{w}^{2})+ \\ \alpha_{1}\left[\sum_{v\neq w} \sum_{u\neq w} \left\{(\tau_{uv}A_{uv}-\alpha_{2}t_{u}t_{v})\cdot\delta(l(u),l(v))\right\}+2\sum_{u=1}^{n} \left\{(\tau_{uv}A_{uw}-\alpha_{2}t_{u}t_{v})\cdot\delta(l(u),l(w))\right\}-(0-\alpha_{2}t_{w}^{2})\right] \\ =\sum_{v\neq w} \sum_{u\neq w} \left\{(A_{uv}-\lambda k_{u}k_{v})\cdot\delta(l(u),l(v))\right\}\text{+}\lambda k_{w}^{2}+\alpha_{1}\sum_{v\neq w} \sum_{u\neq w} \left\{(\tau_{uv}A_{uv}-\alpha_{2}t_{u}t_{v})\cdot\delta(l(u),l(v))\right\}+\alpha_{2}t_{w}^{2} \\ 2\underbrace{\left(\sum_{u=1}^{n} A_{uw}\delta(l(u),l(w))-\lambda k_{w}K_{l}+\alpha_{1}\left\{\sum_{u=1}^{n} \tau_{uv}A_{uv}\delta(l(u),l(w))-\alpha_{2}t_{w}T_{l}\right\}\right)}_{\text{ related to the '}l(w)\text{'}} \end{matrix}$. (S-6)

Same with detecting community, optimizing the function H_h_ is an NP-hard problem. Therefore, we adopt the greedy strategy to solve the problem. According to the terms related to the ‘*l*(w)’ in Eq. (S-6), we can greedily optimize the function H_h_ from the perspective of each node and the update rule of LPAh is formulated as:

$l'(v)=\mathop{\mathrm{argmax}}\limits_{l\in L} (\sum_{u=1}^{n} (1+\alpha_{1}\tau_{uv})A_{uv}\delta(l(u),l)-\lambda k_{v}K'_{l}-\alpha_{1}\alpha_{2}t_{v}T'_{l})$, (S-7)

where, $K'_{l}$ and $T'_{l}$ represent that the old label of node *v* should be ignored, and they can be formulated as:

$K_{l}^{'}=\left\{\begin{matrix} K_{l} ,l\neq l(v) \\ K_{l}-k_{v},l=l(v) \end{matrix}\right.$, (S-8)

$T'_{l}=\left\{\begin{matrix} T_{l} ,l\neq l(v) \\ T_{l}-t_{v},l=l(v) \end{matrix}\right.$. (S-9)
